# Thermoelectric Properties of One-Pot Hydrothermally Synthesized Solution-Processable PEDOT:PSS/MWCNT Composite Materials

**DOI:** 10.3390/polym15183781

**Published:** 2023-09-15

**Authors:** Haibin Li, Shisheng Zhou, Shanxiang Han, Rubai Luo, Jingbo Hu, Bin Du, Kenan Yang, Yizhi Bao, Junjie Jia, Xuemei Zhang

**Affiliations:** 1 School of Mechanical and Precision Instrument Engineering, Xi’an University of Technology, Xi’an 710048, China; lhbzwt@163.com (H.L.); 1200210002@stu.xaut.edu.cn (K.Y.); 2 Faculty of Printing, Packaging Engineering and Digital Media Technology, Xi’an University of Technology, Xi’an 710048, China; s.x.han@stu.xaut.edu.cn (S.H.); hujingboxaut@163.com (J.H.); dubin@xaut.edu.cn (B.D.); baoyizhi0424@163.com (Y.B.); 13429762525@163.com (J.J.); 17791496292@163.com (X.Z.); 3 Shaanxi Provincial Key Laboratory of Printing and Packaging Engineering, Xi’an University of Technology, Xi’an 710048, China; 4 Shanxi Key Laboratory of Advanced Manufacturing Technology, North University of China, Taiyuan 038507, China

**Keywords:** PEDOT:PSS/MWCNTs, thermoelectric ink, DIW printing

## Abstract

The combination of organic and inorganic materials has been considered an effective solution for achieving ambient thermoelectric energy harvesting and has been developing rapidly. Here, PEDOT:PSS/MWCNT (PPM) composite hydrogels were synthesized using the self-assembled gelation process of poly(3,4-ethylenedioxythiophene)-poly(styrenesulfonate) (PEDOT:PSS) and the interaction between PEDOT:PSS and multi-walled carbon nanotubes (MWCNTs) without the addition of any surfactant. After immersion in dimethyl sulfoxide and freeze-drying, the hydrogel is easily dispersed in water and used as a direct ink writing (DIW) 3D printing ink. At room temperature, the PPM-20 printed film with 20 wt% MWCNT solids achieved a maximum power factor of 7.37 μW m^−1^ K^−2^ and maintained stable thermoelectric properties during repeated bending cycles. On this basis, a thermoelectric generator (TEG) consisting of five legs was printed, which could be produced to generate an open circuit voltage of 6.4 mV and a maximum output power of 40.48 nW at a temperature gradient of 50 K, confirming its great potential for application in high-performance flexible organic/inorganic thermoelectric materials.

## 1. Introduction

Thermoelectric generators (TEGs) realize the conversion between thermal energy and electrical energy through the Seebeck effect, especially flexible TEGs, which have broad application prospects in the fields of flexible electronics, medical monitoring, and the Internet of Things by utilizing their waste heat recovery capabilities [1,2,3]. As the core component of TEGs, the types of thermoelectric materials include inorganic semiconductors, organic carbon materials, and conductive polymers [4,5]. The thermoelectric properties of materials are usually evaluated by the dimensionless figure of merit (ZT), ZT = S^2^ σT/k, where S, σ, T, and k stand for the Seebeck coefficient or thermopower, electrical conductivity, absolute temperature, and thermal conductivity, respectively. Compared with conventional inorganic thermoelectric materials, conducting polymers (CPs) have attracted extensive attention in the field of flexible electronics due to their inherent low cost, flexibility, and excellent solution processability [6,7]. Meanwhile, with the large-scale application of additive manufacturing technology, the demand for organic CPs materials is gradually increasing. Among them, the conjugated conductive material PEDOT:PSS exhibits high thermoelectric properties, low thermal conductivity, and a highly adjustable molecular structure or composition, which is considered one of the best candidates for organic thermoelectric materials. In particular, PEDOT:PSS-based thermoelectric materials have proven their ability to more efficiently fabricate high-performance flexible TEGs through additive manufacturing techniques [8,9,10].

In previous studies, in order to improve the carrier transport property and enhance thermoelectric performance, poly(3,4-ethylenedioxythiophene)-poly(styrenesulfonate) (PEDOT:PSS) has always been doped or post-treated with the help of organic solvents, acid or base solutions, and ionic liquids [11,12,13]. However, the low thermoelectric performance of the doped PEDOT:PSS is a key drawback for its use in practical applications. Therefore, the incorporation of nanomaterials into PEDOT:PSS has been demonstrated as an effective method to realize enhanced thermoelectric performance, which not only maintains a high σ value but also improves the S value [14]. In particular, there is a strong π–π interaction between carbon-based nanomaterials and PEDOT:PSS, which could reduce conjugated defects and lower the carrier hopping barrier. PEDOT:PSS/Carbon nanotubes (CNTs) composites are considered a promising strategy to enhance the thermoelectric properties of composites, and PEDOT:PSS aqueous solution can reduce the physical entanglement of CNTs [15,16,17,18]. For instance, Wei et al. [19] fabricated SWCNTs/PEDOT:PSS composite thermoelectric films by vacuum filtration combined with post-treatment and designed a TEG with an S-shaped architecture. After ionic liquid treatment, the conductivity was increased to 1562 ± 170 S cm^−1^, and the Seebeck coefficient remained constant at 21.9 μV K^−1^. He et al. [20] reported a ternary stretchable and flexible thermoelectric film based on PEDOT:PSS/CNT/WPU with Seebeck coefficient and conductivity of 31 μV K^−1^ and 18 S cm^−1^ at room temperature, respectively. The double-network conductive bridge composed of PEDOT:PSS and CNTs acts as both a thermoelectric material and a temperature and strain sensing medium, and the assembled sensor successfully detected temperature changes and strain deformation under self-powered conditions. However, most of the previous studies were mainly focused on the direct mixing of PEDOT:PSS/CNTs and further preparation of TEG by thin film techniques such as suction filtration and drop casting. Meanwhile, in a previous study, highly conductive PEDOT:PSS hydrogel fibers were successfully obtained through a sulfuric acid-assisted gelation process [21]. This inspired us to explore whether CNTs can be used as a reinforcing agent to composite with PEDOT:PSS through a gel process. To the best of our knowledge, no one has systematically investigated the thermoelectric properties of inks converted from self-assembled PEDOT:PSS/CNTs composite gels. 

Compared with simple thin film preparation methods, additive manufacturing has unique advantages such as simple operation, high flexibility, and implementation of complex structures, and this technology is widely selected for the manufacture of new flexible devices [22,23]. Among them, direct ink writing (DIW) technology has low requirements for ink and is widely used in flexible sensors, robots, and biomedical equipment [24]. In this study, we report a simple one-pot hydrothermal method that allows the self-assembly of PEDOT:PSS solution into a three-dimensional hydrogel at an acidic environment. During this process, the added MWCNTs were uniformly and tightly bridged onto the PEDOT:PSS framework. The resulting hydrogel could be easily dispersed in water after freeze-drying and used as extrusion-based 3D printing ink. Compared to previous studies on the direct composites of PEDOT:PSS and MWCNTs for the preparation of thermoelectric inks, we propose for the first time the successful incorporation of MWCNTs into the PEDOT:PSS matrix during the gel formation process, resulting in a high-performance thermoelectric material that can be processed via solution methods. During the gel self-assembly formation process, the PEDOT chain coil conformation was transformed and PSS was partially removed from the PEDOT:PSS complex, which significantly improved the electrical conductivity of the inks. The introduction of MWCNTs and post-treatment with organic solvents greatly enhance the thermoelectric properties of the resulting composite thermoelectric ink. Among them, the PEDOT:PSS/MWCNT (PPM-20) composite ink, containing 20 wt% MWCNTs, exhibited good electrical conductivity and Seebeck coefficient when drop cast to prepare thermoelectric thin films, and could also withstand significant bending. Additionally, the performance of TEG prepared using DIW process was also investigated in this paper.

## 2. Materials and Methods

### 2.1. Materials

The PEDOT:PSS aqueous solution (Clevios PH1000, PSS:PEDOT = 2.5:1) was obtained from Germany Heraeus Co., Ltd. MWCNTs (Hanau, Germany) (purity higher than 85%, diameter of 5–8 nm) were purchased from China Macklin Co., Ltd. (Shanghai, China). All other reagents including dimethyl sulfoxide (DMSO) and sulfuric acid (H_2_SO_4_) were of laboratory grade (Macklin Co., Ltd., Shanghai, China). All the materials were used without further purification.

### 2.2. Fabrication of PEDOT:PSS/MWCNT Composite Aerogels, Inks, and Thermoelectric Films

The different PEDOT:PSS/MWCNT composite thermoelectric inks were obtained by dispersing self-assembled PEDOT:PSS/MWCNT aerogels with different MWCNT contents. Among them, the prerequisites for preparing PEDOT:PSS/MWCNT aerogels is to synthesize the composite hydrogels using a one-pot hydrothermal method. Firstly, different masses of MWCNTs (0, 1.89, 4, 6.35, 9, 12, 15.4, or 24 mg) were dispersed in PEDOT:PSS dispersion (3 mL) using ultrasonication in an ice bath. After stirring at room temperature for 1 h, 0.05 M H_2_SO_4_ was added to the composite system and the mixtures were further stirred for 10 min. Then, the homogeneous mixtures were transferred to a Teflon liner and subjected to a hydrothermal reaction at 90 °C for 3 h. After cooling to room temperature, the resulting cylindrical composite hydrogels were naturally cooled and soaked in DMSO for 6 h. Subsequently, the treated hydrogels were repeatedly washed with deionized water (DI) and placed in the cold trap of a freeze-dryer at −65 °C for 6 h. After 48 h of vacuum freeze-drying, the PEDOT:PSS/MWCNT hydrogels with different MWCNT contents were successfully transformed into corresponding composite aerogels. Finally, the composite aerogels were dispersed in water with the assistance of ultrasonication to prepare thermoelectric inks with a solids content of 2 wt% and stored in a refrigerator at 4 °C.

To fabricate free-standing films, the ink was drop casted on PET substrates pre-treated with UV/O_3_ for 5 min for better wetting with the aqueous solution. Then, the film was annealed at 130 °C for 30 min and cut to a size of 35 mm × 7 mm for measurement. The thickness of the films, measured by a profilometer (Alpha-Step, KLA Tencor, Milpitas, CA, USA), was in a range between 100 and 200 μm.

### 2.3. Fabrication of Flexible TEG

The flexible TE generators based on PEDOT:PSS/MWCNT composite ink were fabricated by the DIW process. The printing procedure was conducted based on an in-house developed 3D printer, and using Repetier Host application to process the thermoelectric array model convert it into G-code (Figure S1). Five thermoelectric legs were printed on a PET substrate pre-treated with UV/O_3_ using ink dispersion (nozzle diameter: 0.5 mm, print speed: 300 mm/min). After the first printing, thermoelectric arrays were dried on a hotplate at 85 °C, and at 65 °C for the subsequent print layers. To achieve good uniformity and thickness, 7 layers were printed repeatedly. Finally, the thermoelectric legs were connected in series using conductive silver paste, and the whole TEG was dried in an oven at 120 °C for 1 h.

### 2.4. Measurements and Characterizations

The morphologies of the composite aerogels were analyzed by using scanning electron microscopy (SEM Hitachi S4700, Tokyo, Japan) at an acceleration voltage of 15 kV. Fourier transform infrared spectroscopy (FT-IR) was collected on an IR-spirit FTIR spectrometer (Shimadzu, Kyoto, Japan) using attenuated total reflection (ATR) mode from 4000 to 400 cm^−1^ with 20 scans. X-ray photoelectron spectroscopy (XPS) spectra was recorded on an AXISULTRA spectrometer (Kratos, Manchester, UK) with monochromatic Al Kα (1486.71 eV) line at a power of 100 W (10 mA, 10 kV). The measurement of electrical conductivity was carried out using four-probe measuring instrument (Four Probe Technology RST-9, Guangzhou, China). The Seebeck coefficients of all samples were measured using a custom-made system consisting of a digital source meter (Keithley-2460, Tektronix, Beaverton, OR, USA), a controlled Peltier heater, a type K thermocouple, and a power supply. The Seebeck coefficient was determined by linearly fitting ΔV/ΔT to ΔV values measured at ten different ΔT values, taking five data points at each temperature. The measured absolute Seebeck coefficients (20.2 ± 0.4 µV K^−1^ at 20 °C) of pure nickel foil were used for calibration. The output performance of the TEG was tested at a temperature difference of 50 K by connecting a variable resistance box in series, where current and voltage values were obtained from a series ammeter and a parallel voltmeter, respectively.

## 3. Results and Discussion

**Figure 1:**
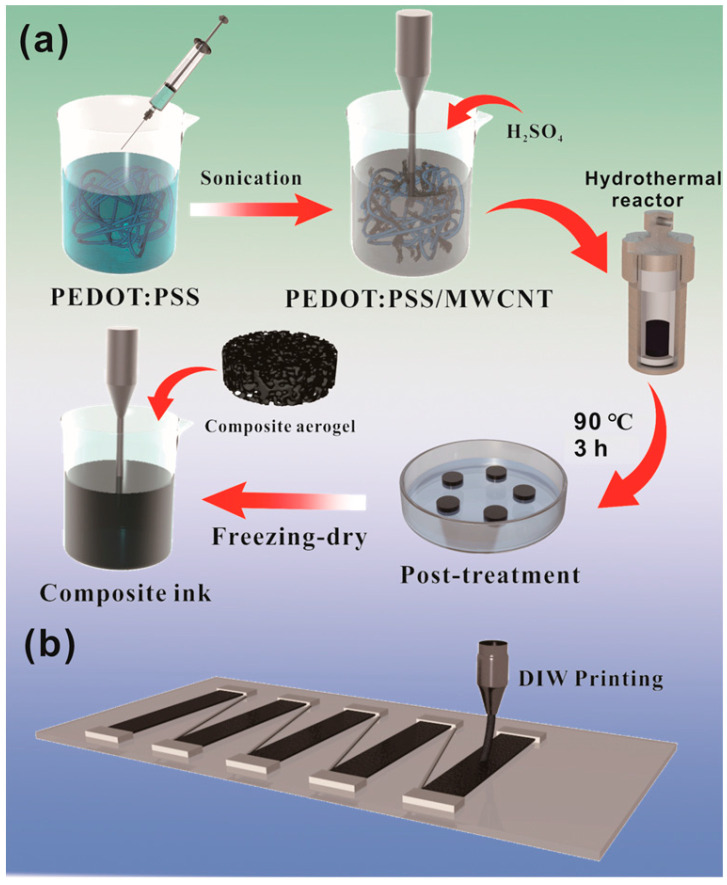
Schematic illustration of (**a**) the preparation of the PPM composite ink and (**b**) the printing process of TEG.

Figure 1a schematically illustrates the manufacturing process of PEDOT:PSS/MWCNT composite inks, which includes the self-assembly gelation, the immersion treatment, the freeze-drying, and the subsequent dispersion process. In brief, the PEDOT:PSS dispersions containing MWCNTs were used as precursors for hydrothermal reactions. With the promotion of H_2_SO_4_, stable composite hydrogels were obtained by using π–π interactions and van der Waals forces between MWCNTs and PEDOT:PSS. The aerogel obtained after further freeze-drying of the hydrogel could be easily dispersed into DI through sonication, and the dispersion was well suitable for the DIW process (Figure 1b). To distinguish them, the composites were referred to as PPM, and according to the mass ratio of MWCNTs (0, 5, 10, 15, 20, 25, 30, and 40 wt%) in in the composite system correspondingly they were denoted as PPM-0, PPM-5, PPM-10, PPM-15, PPM-20, PPM-25, PPM-30, and PPM-40. For comparison, the original PEDOT:PSS solution (PH1000) and the PEDOT:PSS gel (PPG) without DMSO treatment were also prepared as control samples. It is noteworthy that composite solutions with a mass ratio of MWCNTs greater than 50 wt% have difficulty in forming stable gels by means of the self-assembly process of PEDOT:PSS, indicating that excessive MWCNT agglomeration disrupts the gel network.

**Figure 2:**
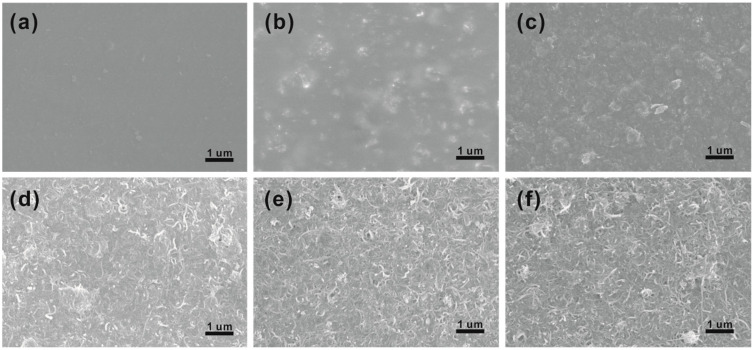
SEM surface images of (**a**) PPM-0, (**b**) PPM-5, (**c**) PPM-10, (**d**) PPM-20, (**e**) PPM-30, and (**f**) PPM-40 films.

Figure 2 displays the morphology of PPM films with different MWCNT contents. It is obvious that the surface of the pure PPM-0 film is homogeneous and flat (Figure 2a). After the introduction of MWCNTs, the surface of the composite film gradually became rough until the interwoven MWCNTs could be clearly observed. When the MWCNT content is below 10 wt%, the few MWCNTs in the composite film were randomly distributed in the PEDOT:PSS matrix, and the less interconnected MWCNTs in PEDOT:PSS may affect the contact resistance between conductive PEDOTs. As the MWCNT content increases to 20 wt%, interwoven network-like structures of MWCNTs were clearly observed on the film surface, which is favorable for improving the Seebeck coefficient. In addition, the surface of MWCNTs is covered by PEDOT:PSS, which facilitates electron carrier mobility between MWCNTs and also results in low contrast of SEM images. However, when the content of MWCNTs was further increased to 40 wt%, some larger agglomerations appeared on the surface of the films, which might affect the electrical conductivity of the composite films.

**Figure 3:**
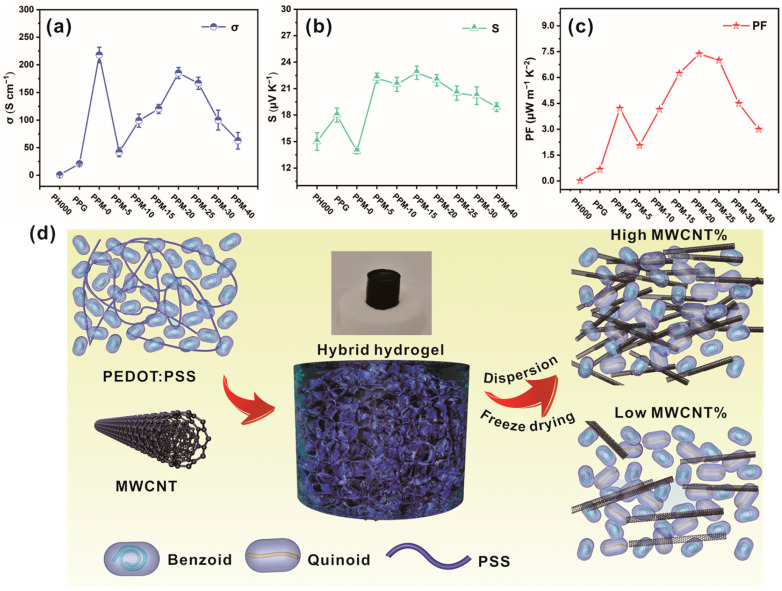
Room-temperature TE performances of all samples including (**a**) electrical conductivity, (**b**) Seebeck coefficient, and (**c**) power factor. (**d**) Conceptual illustration of the distribution of PEDOT:PSS and MWCNTs in different periods of composite morphology.

Figure 3 shows the TE properties of the prepared PPG and PPM composite films with different MWCNT concentrations. As shown in Figure 3a, compared with the original PEDOT:PSS, the gel formation process, the introduction of MWCNTs and the treatment of the gel with DMSO immersion are effective in improving the electrical conductivity of the film. In particular, the electrical conductivity of PPM-0 films prepared using PEDOT:PSS hydrogels treated with DMSO was significantly increased to 218 S cm^−1^. This apparent enhancement is attributed to the increased carrier mobility induced by the removal of PSS and the transformation of the PEDOT laminar structure. This is because in the original PEDOT:PSS, there is only one bond (C-C) between the carbon atoms of the EDOT monomer benzene ring, which corresponds to the coil-like benzene-fused structure. After the self-assembled gelation process and post-treatment with DMSO, the randomly coiled benzene-fused structure transforms into an extended quinoid structure (C=C) (Figure 3d) [25]. This transformation allows the formation of longer chains with stronger electron affinity and better conjugation properties, which can enhance the conductivity of PEDOT. Additionally, the formation of quinoid structure in PEDOT:PSS materials also contributes to improving the stability and durability of the material. Despite its excellent conductivity performance, the Seebeck coefficient of the post-treated PPM-0 film was significantly decreased. The conductivity of the PPM composite films decreased and the Seebeck coefficient increased after the addition of 5 wt% MWCNTs. This is due to the decrease in carrier concentration caused by the energy filtering effect between the n-type MWCNT and the p-type PEDOT:PSS interfaces, where the few and discontinuous MWCNTs act as resistive junctions in the highly conductive PEDOT:PSS. As illustrated in Figure 3d, carrier transport at low MWCNT concentrations occurs through the MWCNT-PEDOT:PSS–MWCNT junction. With the increase in the MWCNT content from 5 wt% to 20 wt%, the conductivity of the PPM composite film consistently increased to 185.19 S cm^−1^ due to a gradual reduction in the internal PEDOT:PSS–MWCNT junction, allowing for a more continuous conductive pathway. However, in the case of high MWCNT content, the conductivity tends to decrease because of the excessive MWCNT self-aggregation and excessive coverage of PEDOT:PSS, which is consistent with what was observed in the SEM images. Most notably, the Seebeck coefficient and electrical conductivity of the PPM composite films do not exhibit a monotonic relationship; that is, (i.e., the values of S and σ show an opposite trend). As shown in Figure 3b,c the addition of a small amount of MWCNTs can cause a significant increase in the Seebeck coefficient of the PPM composite film. The interfacial energy filtering effect between the two materials allows the passage of high-energy carriers and enhances the interfacial density [26]. The highest Seebeck coefficient of 22.8 μV K^−1^ has been achieved for the PPM-15 composite film, which is more than two times higher than that of PPM-0. As the concentration of MWCNTs increases, the n-type thermoelectric properties may intensify the phonon-polariton interaction, and the Seebeck coefficient of the composite film decreases slightly. The previous studies have indicated that the phonon-polaron interaction leads to scattering processes in electron transport, thereby affecting the magnitude of the Seebeck coefficient. Enough MWCNTs promote the propagation of phonons while also causing reverse scattering of low-energy and high-energy charge carriers, resulting in a shorter average free time for electron transport and a smaller Seebeck coefficient [27]. Combined with conductivity and Seebeck coefficient analysis, the PPM-20 composite film exhibited the highest power factor (PF = S^2^σ) of 7.37 μW m^−1^ K^−2^. According to previous studies, the power factor of the directly dispersed PEDOT:PSS/MWCNT composite was only 0.09 [28]. It is noteworthy that the PEDOT:PSS in all of the prepared PPM composites was not doped beforehand. This means that the PPM composite films obtained by gelation, immersion, and then cold-dry dispersion have great potential for TE applications, and more studies are still needed to further understand this point.

**Figure 4:**
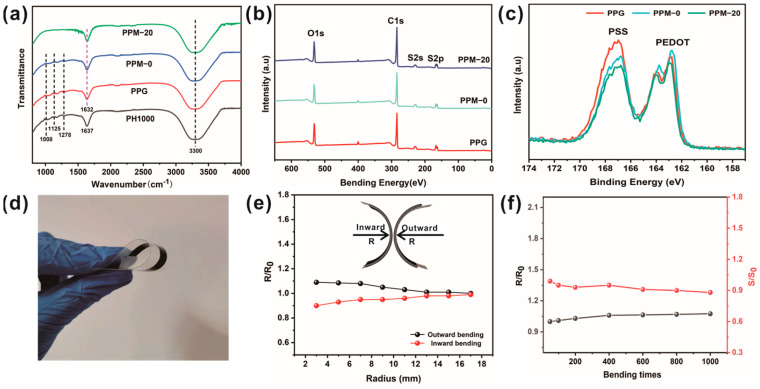
(**a**) FTIR spectra of pure PEDOT-PSS, PPG, PPM-0, and PPM-20. (**b**) XPS full spectra and (**c**) S_2p_ spectra of PPG, PPM-0, and PPM-20. (**d**) Digital photos of flexible PPM-20 composite film. (**e**) The relative change in resistance for different inward or outward bending radiuses. (**f**) The relative change in conductivity and Seebeck coefficient at different bending cycles for a bending radius of 7 mm.

In order to elucidate the mechanism of the enhanced thermoelectric properties of PPM composite materials, Fourier infrared spectroscopy and XPS techniques have been employed. As shown in Figure 4a, in the spectrum of the CNT-free ink, the band at 1278 cm^−1^ is due to the C-C stretching vibration of the thiophene ring. The peaks at 1008 and 1125 cm^−1^ are assigned to the asymmetric vibration of S-O in PSS macromolecules and sulfonate groups, respectively. Meanwhile, the spectra of inks exhibit a strong broad band at 3300 cm^−1^, which is caused by the O-H stretching modes of the hydroxyl groups, and this peak gradually increases with the superposition of the treatments [29]. Compared to PH1000, the peak of C=C stretching of quinoid structure at 1637 cm^−1^ red-shifts to 1632 cm^−1^ in PPG and PPM ink, which indicates the transformation of the benzoid structure of PEDOT:PSS to quinone structure during gelation [30]. In addition, from the FT-IR spectra of PPM-20 composite ink, some of the characteristic peaks of PEDOT:PSS are masked, indicating a strong interaction between PEDOT:PSS and MWCNTs.

XPS analysis was used to investigate the change in the PSS component during the gelation process and subsequent solvent post-treatment process. Figure 4b,c show the full spectra and S_2p_ XPS spectra of the PPG, PPM-0, and PPM-20 films, which reveal that the thermoelectric films are composed mainly of elements C, S, and O. In Figure 4c, the peak at 170–166 eV is associated with the sulfur atom of the sulfonic acid group in PSS chains [31]. While the two peaks at 162–166 eV are caused by the spin-orbit coupling of the C-S bond of the thiophene ring in the PEDOT chains, the peak areas are usually used to estimate the relative amounts of PEDOT and PSS [32]. Compared to the original PH1000 (2.0), the PSS and PEDOT ratios for PPG, PPM-0, and PPM-20 were 1.39, 1.26, and 1.21. These results demonstrate that the self-gelation process and post-treatment are effective in removing PSS, resulting in an increase in PEDOT-rich domains. This leads to a further enhancement of the thermoelectric properties. In particular, the screen effect of the DMSO bath can substantially reduce the Coulombic forces between PEDOT and PSS, which is consistent with the trend of thermoelectric properties in Figure 3. In addition, given the further decrease in the PSS/PEDOT ratio of PPM-20, the π–π interaction between MWCNTs and PEDOT:PSS also induces phase separation to some extent during the self-gelling process, resulting in an ordered lamellar structure that is more favorable for the transport of charge carriers.

As shown in Figure 4d, PPM-20 thermoelectric films exhibit favorable flexibility. Considering the stability required in the practical application of flexible thermoelectric components, we further investigated the TE performance of the PPM-20 composite film during bending (Figure 4e,f). The front and back of the film are mounted on separate cylinders surfaces of different radii so that they undergo different degrees of inward and outward bending. The results suggest that the film presents only a small increase or decrease in resistance, even at a bending radius of 3 mm. Furthermore, after as many as 1000 bends, the resistance and Seebeck coefficient of the PPM-20 film did not change significantly, displaying excellent stability and durability, which is beneficial for practical applications.

**Figure 5:**
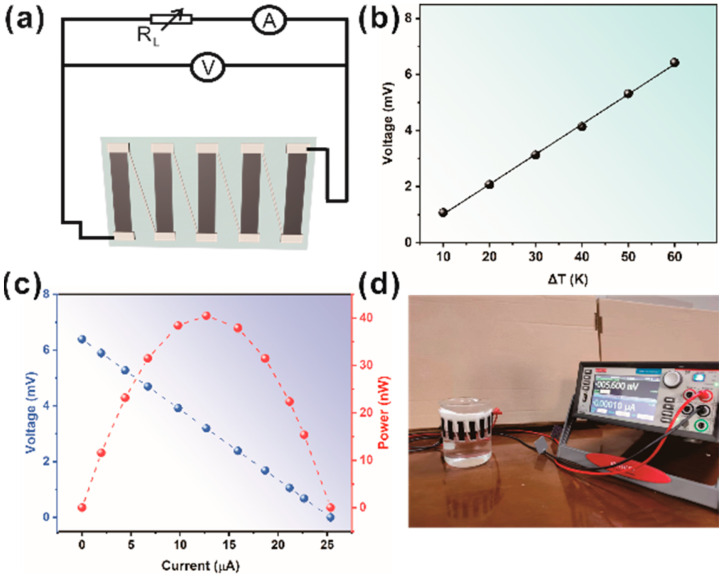
(**a**) Schematic diagram of the circuit for evaluating the output characteristics of the five-leg TEG. (**b**) The relationship between the output voltage and temperature gradient with five legs. (**c**) Voltage–current–power output curves at ΔT = 50 K. (**d**) Photograph of the 5.6 mV voltage created when the TEG was wrapped around a beaker and 70 °C warm water was poured in until it touched the underside of the TEG.

In order to test the power generation characteristics of the PPM-20 thermoelectric material, five thermoelectric legs were printed on the PET substrate using the DIW process. They were connected in series with silver paste to form a TEG (Figure 5a). Figure 5b shows a sketch of the circuit schematic assembled to measure the performance of the TEG, in which the load resistance is controlled by an adjustable resistor module. It can be seen from Figure 5c that the open-circuit voltage generated by the TEG increases almost linearly with the temperature gradient (ΔT = 0–50 K). In addition, the output voltage, output current, and output power of the TEG were measured with different external load resistors at a stable temperature difference of 50 K (Figure 5c). The output power of the TEG can be determined using the following equations:

(1) $$P={\left(\frac{U}{R_{in}+R_{load}}\right)}^{2}R_{load}$$

where *R_in_* is internal resistance and *R_load_* is load resistance. As the external load resistance varies, the output voltage and current are inversely proportional, and the output power exhibits a parabolic relationship with the current. TEG produces a maximum output power of 40.48 nW when the load resistance is equal to the internal resistance of the TEG. Moreover, as shown in Figure 5d, the beaker with the TEG attached was continuously poured with 70 °C hot water and reached the lower edge of the TEG, producing a potential difference of 5.6 mV. This indicates that the PPM composite film can effectively trap waste heat from the environment. For wider commercial applications, the performance of PPM-20 thermoelectric devices should be further improved. Doping modifications to PEDOT:PSS was able to substantially improve the electrical properties, which may have a direct impact on the thermoelectric properties of the PPM composite inks.

## 4. Conclusions

In summary, a simple one-pot hydrothermal method has been developed for the synthesis of solution-processable PPM composites. During the self-assembly of the gel, MWCNTs are firmly bridged to the PEDOT:PSS three-dimensional backbone due to π–π interactions and van der Waals forces between PEDOT:PSS and MWCNTs. The PPM aerogel obtained by freeze-drying is well dispersed in water and has been proven to be suitable for use in the DIW process. The energy filtering effect induced by the introduction of a small amount of MWCNTs in the composite system filters the low energy carriers and improved the Seebeck coefficient. The PPM-20 composite film showed a maximum PF value of 7.37 µW m at a solid content of MWCNTs of 20 wt%. In addition, the printed PPM-20 film has good flexibility and stable thermoelectric properties, which can withstand a wide range of repetitive bending variations. Finally, a TEG consisting of 5 legs manufactured by the fully printed process generated a maximum output power of 40.48 nW at a temperature difference of 50 K. This method of synthesizing solution-processable PEDOT:PSS/MWCNTs inks by self-assembly has great potential to facilitate the development and application of printable organic/inorganic composite thermoelectric materials.

## Data Availability

The datasets used and analyzed in the current study are available from the corresponding author on reasonable request.

## Supplementary Materials

The following supporting information can be downloaded at: https://www.mdpi.com/article/10.3390/polym15183781/s1, Figure S1: Photos of a self-made 3D printing extrusion system and Repetier Host application interface.
