# Supplementary material for: Thermoelectric Properties of One-Pot Hydrothermally Synthesized Solution-Processable PEDOT:PSS/MWCNT Composite Materials
Source: Polymers (Basel). 2023 Sep 15;15(18):3781. doi: 10.3390/polym15183781 (PMC10534393; doi:10.3390/polym15183781)
Supplement: Supplementary file 1 [file polymers-15-03781-s001.zip › polymers-2591507-supplementary.pdf]

Thermoelectric properties of one-pot hydrothermally synthesized solution-processable PEDOT:PSS/MWCNTs composite materials

Haibin Li [1], Shanxiang Han [1], Ruibai Luo [2,4,*], Jingbo Hu [2,4], Bin Du [2,3], Kenan Yang [1], Shisheng Zhou [2,3,*], Yizhi Bao [2], Junjie Jia [2] and Xuemei Zhang [2]

[1] School of Mechanical and Precision Instrument Engineering, Xi’an University of Technology, Xi’an 710048, China; lhbzwt@163.com (H.L.); 1200210002@stu.xaut.edu.cn (K.Y.).

[2] Faculty of Printing, Packaging Engineering and Digital Media Technology, Xi’an University of Technology, Xi’an 710048, China; s.x.han@stu.xaut.edu.cn (S.H.); baoyizhi0424@163.com (Y.B.); 13429762525@163.com (J.J.); 17791496292@163.com (X.Z.).

[3] Shaanxi Provincial Key Laboratory of printing and Packaging Engineering, Xi’an University of Technology, Xi’an 710048, China; dubin@xaut.edu.cn (B.D.).

[4] Shanxi Key Laboratory of Advanced Manufacturing Technology, North University of China, Taiyuan 038507, China; hujingboxaut@163.com (J.H.).

[*] Correspondence: zhoushisheng@xaut.edu.cn (S.Z.); luorubai@xut.edu.cn (R.L.)

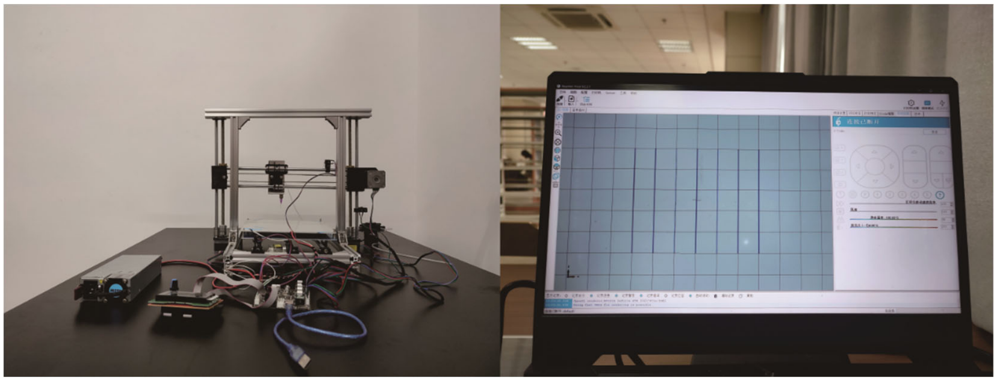

Figure S1. Photos of a self-made 3D printing extrusion system and Repetier Host application interface.
